# COVID-19: Threat or Opportunity for the Portuguese Higher Education's Attractiveness for International Students?

**DOI:** 10.1177/10283153221121396

**Published:** 2023-02

**Authors:** Cristina Sin, Orlanda Tavares, Joyce Aguiar

**Affiliations:** 1 CeiED (Interdisciplinary Research Centre for Education and Development), Universidade Lusófona, Lisbon, Portugal and CIPES - Centre for Research in Higher Education Policies, Matosinhos, Portugal; 2 CIPES - Centre for Research in Higher Education Policies, Rua 1° de Dezembro 399, 4550-227 Matosinhos, Portugal

**Keywords:** international recruitment strategies, international student support, COVID-19, Portugal

## Abstract

The paper analyses the influence of COVID-19 on Portuguese institutions’ intake of international students and their responses to the pandemic. Two dimensions are considered: quantitative impact on international enrolments and higher education institutions’ strategies for the recruitment and support of international students. The first dimension is analysed through national statistics and comparison of enrolments over the past five years. Then, the institutional strategies implemented to encourage recruitment of new international students and to support existing ones during the lockdown are explored. Statistics show that COVID-19 had a negative impact on international enrolments, although less severe than expected. Growth has continued, but at a much slower pace than in the past few years. The slowdown in growth was much more pronounced in polytechnics than in universities. Additionally, the measures implemented by Portuguese higher education institutions suggest that these have coped with COVID-19 as an opportunity to rethink and redefine strategies.

## Introduction

The coronavirus (COVID-19) pandemic has taken the world by surprise and represents an unprecedented challenge for all sectors of the society, including students and higher education institutions. In Portugal the first two cases of COVID-19 were identified on 2 March 2020. About two weeks later, the Portuguese Government declared a state of emergency, which triggered the closure of all schools, kindergartens, shops and business in general, except those considered essential (DRE, 2020a). Nevertheless, a few days before the state of emergency, the Ministry of Science, Technology and Higher Education (MCTES) had already announced the decision to close all higher education institutions (HEIs). It strongly recommended ‘online teaching and learning, keeping the activities through teacher and student interaction via digital tools’ (MCTES, 2020). On 2 May 2020, the unlocking period began, and the COVID-19 restriction measures were gradually alleviated (DRE, 2020b). Schools reopened in June – only for students in 11^th^ and 12^th^ grades to prepare for entrance exams – while HEIs continued online teaching and learning whenever possible, until the end of the 2019/20 academic year.

For the 2020/21 academic year, the MCTES stipulated that HEIs had to ensure ‘preventive conditions, in a context in which the combination of face-to-face teaching with other forms of teaching and learning would continue, involving, in particular, distance learning’ (Direção-Geral do Ensino Superior [DGES], 2020). To meet the need for a sudden shift to distance education, it launched the “Skills 4 post-COVID - Skills for the Future” programme, aiming to promote the rapid implementation of new approaches to teaching, learning and research. One of the expected results of this programme was fostering ‘the attraction of international students, providing and promoting *COVID-free* conditions in HEIs’ (DGES, p.4, 2020).

In November 2020, the number of COVID-19 cases and deaths increased very rapidly across the country, indicating a new pandemic wave. On 14 January 2021, the Council of Ministers announced a new state of emergency and the suspension of classroom teaching and non-teaching activities, as part of extraordinary measures to mitigate the epidemiological situation. The state of emergency was repeatedly renewed until April 2021, when HEIs gradually started to reopen in conditions similar to those before the lockdown, i.e. blended learning and teaching. Face-to-face activities at HEIs are now allowed, as well as attendance to carry out assessments and professional activities, although teleworking and online classes are still encouraged. In addition, the classes are divided into two groups, which alternate weekly or every two weeks between the face-to-face and online lessons.

The general measures adopted by institutions around the world to cope with the COVID-19 emergency and to support students have been rather well documented (Agasisti & Soncin, 2021; de Boer, 2021; Pham & Ho, 2020; Yang & Huang, 2021): adoption of distance or blended learning, training students and teaching staff in digital competences, offering IT equipment to students, student psychological support, reduction of tuition fees, postponing exams, etc. However, to the best of our knowledge, there is hardly any research into how HEIs have acted to support international students. These students represent a particularly vulnerable group who is expected to suffer most from the consequences of lockdowns and institution closures, because they are away from their home country and because, during this period, travel restrictions were frequently in place. Surveys conducted to find out about prospective international students’ plans (BridgeU, 2020; Studyportals, 2020) suggest that a considerable number intend to change their study plans, either by postponing enrolment by up to one year, choosing to study in their home country or changing country. They are also more likely to study closer to home (Dennis, 2020).

Additionally, a reshuffling of international destinations appears underway (Bista et al. 2021). The pandemic burst out in a time of already changing trends in student mobility. The dominant flow of international mobility, whereby students come from a variety of countries, mostly Asian ones, and move mainly towards English-speaking countries’ (Börjesson, 2017), has been shifting. Anglo-Saxon countries have weakened their share as the major recruiters, while other countries are becoming popular international student destinations (Perkins and Neumayer, 2014). ‘Brexit’ and the election of Trump, and subsequent aggressive anti-immigration policies in the UK and the US, have had a discouraging effect on students, negatively affecting their perceptions of safety, post-graduation work, and immigration opportunities (Choudaha, 2017; Peters et al., 2021; Weimer and Barlete, 2020). Alongside, Asia is becoming a significant importer of mobile students (Bista et al., 2018). The establishment of the European Higher Education Area has allowed smaller countries like Portugal to recognise the importance of international student recruitment and developed strategies accordingly (Sin et al., 2021).

Covid-19 has further contributed to this changing wave. Due to border closures, higher education institutions from major destination countries, such as the US, Canada and Australia, experienced declines in incoming international students, although less severe than expected and interest appears to be growing again for these study destinations (Buckner et al., 2021). But during the pandemic, many Chinese students changed their study-abroad plans for safety reasons and travel restrictions (Mok et al., 2021). Several higher education institutions in the East Asian region have grasped this opportunity and have adopted different strategies to attract Mainland Chinese students who planned to study abroad (Mok et al., 2021). The extent to which the pandemic has affected countries and regions has also influenced student mobility flows (Mok et al., 2021). For instance, Marginson (2020) argues that countries with a better pandemic control will improve their comparative advantage, as questions of health care and health security will weigh more heavily when families and students make decisions about studying abroad.

It thus appears that a new trend is on the rise: regionalization, during and after COVID-19. Mok et al. (2021) argues that this trend will occur in Asia and other regions, such as Europe, where further regionalisation of higher education might intensify. Additionally, some studies are now beginning to examine the growing phenomenon of South-South student migration, instead of just South–North or North-North dynamics (França and Cairns, 2020) and relating it to colonial ties (African Portuguese speaking countries and Brazil). For institutions in new destinations, the pandemic may therefore represent not only a threat, but also an opportunity. To grasp it, HEIs need to pay special attention to the needs of this student group (Sahu, 2020).

Portugal has traditionally received degree-mobile students from Portuguese-speaking countries, but has also become increasingly attractive for students from other countries recently (Sin et al., 2021). The question now becomes how to maintain this growth by adjusting to the current circumstances. This paper aims to analyse whether Portuguese institutions are coping with the pandemic as a threat or as an opportunity. It will consider two aspects: first, the impact of COVID-19 on international student enrolments in Portugal by resorting to national statistics and comparing enrolments between 2019/20 and 2020/21. The focus here lies exclusively on students who are enrolled in a degree course (as opposed to exchange students). Second, it aims to explore the institutional strategies implemented to sustain recruitment of international students and to support existing international students during the lockdown. The observed evolution may be related to students’ negative or positive assessment of the Portuguese management of the crisis and of the institutions’ sensitivity to the needs of international students. According to Tran (2020, xii), “the connection between university communities and international students is more critical than ever”, needing to ensure both learning and wellbeing, because this will make a difference for countries’ and universities’ sustainable international recruitment and reputation as a study destination.

## Mitigating the Effect of COVID-19 on International Students

Although not representing a high-risk group in terms of getting severely ill of COVID-19, students across the world have felt acutely the impact of the lockdown, in general, and university closures, in particular. Research has highlighted the multiple negative effects that the COVID-19 pandemic has had on students at various levels: academic study and performance, mental health, financial situation and social life. Regarding academic performance, the new teaching environment characterised by online learning and/or blended learning presented challenges. While some students reported deficient computer skills, others experienced increased workloads (Aristovnik et al., 2020) and delays in the academic activities and progression (de Boer, 2021). Study delays were caused by the postponing of tests and the cancellation of practical training, work placements or clinical internships in health programmes, causing uncertainties about course progression and completion (Carolan et al., 2020). Similar delays occurred in research (affecting doctoral students), since the lockdown and institutional closures made laboratory work, clinical trials or fieldwork difficult or impossible (van Schalkwyk, 2021). Students’ psychological well-being was also affected, as these were reported to experience stress, depression, anxiety, anger and frustration (Aristovnik et al., 2020; Cao et al., 2020; Odriozola-Gonzáleza et al., 2020). Especially students who were living by themselves were more exposed to social isolation and mental health disorders (Elmer et al., 2020). Social life suffered further to social distancing, the impossibility of meeting with friends, travelling or engaging in social activities (Elmer et al., 2020). A deterioration of students’ financial situation has been another severe consequence of the COVID-19 pandemic, which in most cases is related to the loss of student jobs, and has also triggered worries about the future in general and their career prospects (Aristovnik et al., 2020; Le, 2021).

Although various studies have already considered the impact of the pandemic on students in general, international students have been less an object of research. The experience of international students is, by itself, tough because of the new life circumstances and challenging financial condition they often experience in their study destinations. The extreme situation caused by the COVID-19 crisis has turned them into a particularly vulnerable group (Gallagher et al., 2020; Sahu, 2020). Away from their families and support networks, the shutdown of institutions, the general lockdowns imposed in countries around the world and travel restrictions have aggravated their situation, through the loss of their personal and social contacts and monetary problems as a result of extended stays in the destination country (Sahu, 2020). Institutions have had to ensure food, accommodation, and safety services for international students, as well as provide advice. Sahu (2020) recommended more attention and systematic support to vulnerable international students, for instance by maintaining residences open for students who are unable to return to their homes or even considering financial support.

According to Firang (2020), international students are more likely to experience a severe state of anxiety, social and psychological distress. Moreover, many international students come from developing countries in Asia, Africa or Latin America. A survey into the impact of covid-19 on students worldwide (Aristovnik et al., 2020) found large differences in digital skills and the availability of digital equipment between students from developing and developed parts of the world, with African, Asian, and South American students reporting the poorest results. Students in Asia and Africa were also those suffering most financial problems and finding it harder to cope with the effects of the pandemic on their academic work and lives (Aristovnik et al., 2020). Such findings suggest that international students from these regions may experience additional financial hardships and additional challenges in the adaptation to online teaching and learning. In Portugal, for instance, African and Brazilian students have faced a complex set of challenges, from economic hardships, emotional distress and adaptation to online teaching (Malet Calvo et al., 2021).

Worldwide, the coronavirus crisis has impacted every stage of the decision-making journey of international students, who are looking for more information from universities than ever before (QS Quacquarelli Symonds, 2020). To increase their international student recruitment for 2020, and to position themselves for a strong 2021, institutions are taking innovative steps to attract (or maintain) international students: a great increase in online learning, changing deadlines for application intakes, diversifying source countries, changes in language test requirements or discounting fees (QS Quacquarelli Symonds, 2020).

However, the studies which look at measures to support international students (Le 2021; Metcalfe, 2021; Tran, 2020) are still scarce, as the pandemic is recent and has not come to an end. In Australia, a country whose higher education sector is heavily reliant on international students, political authorities showed little sensitivity towards international students, encouraging them to return to their home countries (despite border closures and cases of temporary visas) and made no efforts to assist them in these difficult circumstances (Le, 2021). For instance, these students were excluded from government relief packages (Gallagher et al., 2020). In Canada, immigration authorities facilitated international students’ stay in the country by maintaining their visa eligibility and continuing to count time toward their post-graduation work permits, even if they were studying online and outside of Canada. Since autumn, they were allowed to enter Canada on condition of staying in quarantine for two weeks. In this context, one university offered international students complimentary two-week self-isolation room and board packages (Metcalfe, 2021).

The support higher education institutions offer to their international students is crucial. Alinda (2021) argues that universities need robust, well-defined and effectively implemented support services to attract and retain international students, as these latter consider these services to contribute positively toward their overall success as students. The continued attractiveness of an institution also depends on student satisfaction: according to Mazzarol and Soutar (2002), word-of-mouth referrals and recommendations are one of the most important promotion vehicle institutions can use. Students, therefore, expect to receive effective support services during their studies. This reliance becomes even more acute in times of crisis, as the one generated by the pandemic, with the ensuing social isolation, loneliness, anxiety and other negative effects on mental well-being (Moscaritolo et al., 2022; van de Velde et al., 2021).

Literature on international student support during Covid-19 is scarce. Institutions around the world had to move their support services online, similar to teaching provision. In the US, Veerasamy and Ammigan (2021) describe how one institution implemented and experienced the shift from a traditional face-to-face model to a virtual model of support for international students further to campus shutdowns. They argue for the importance of training support staff on how to deliver online services effectively to a culturally diverse and vulnerable population, especially in emergency situations which imply rapid transition to remote learning. In the same vein, Bouchey et al. (2021) argue that institutions that had invested in remote services before Covid-19 were at an advantage to adopt emergency remote operations and are also likely to adapt faster their support services in response to future challenges. Agasisti and Soncin (2021) report how one Italian university created networking initiatives especially for international students to avoid isolation, through a series of online events ‘E-BUDDY – Events on the screen to break the quarantine’. Another example comes from Saudi Arabia (Alsulami, 2021) where universities offered financial support, housing students in hotels, provided Internet and offered free meals. Charities and individuals also offered support to international students, financial or in-kind.

## Methodology

To answer the research question, a mixed-method approach was followed. First, a descriptive analysis of official data provided by the General Directorate for Education and Science Statistics (DGEEC) was carried out, for the six academic years between 2015/6 and 2020/21 (see Table 1).

**Table 1.** International students enrolled in a degree in Portuguese HEIs.

	2015/16	2016/17	2017/18	2018/19	2019/20	2020/21
**Short technical degree**	143	270	403	1008	2251	2410
**First cycle**	6 715	7 395	10025	12807	16900	18528
**Second cycle**	7 897	8 005	11507	15090	17418	18133
**Third cycle**	4886	4 849	5879	6558	7146	6973
**Postgraduate specialisations**	174	236	308	292	287	225
**Total**	19815	20755	28122	35755	44005	46269

Source: DGEEC, 2021.

The data contains information on all students enrolled in Portuguese HEIs. International students in this analysis were considered to be those indicated in the database as having completed secondary education in a country other than Portugal, but excluding students on credit mobility. In Portugal higher education degrees are aligned to the study architecture recommended by the Bologna Process, which organises programmes into three different levels called cycles. The first cycle corresponds to the bachelor's degree; the second cycle is equivalent to a master's degree, and the third cycle to a doctoral degree. Additionally, higher education institutions offer non-degree awarding short technical degrees lasting two years, which grant access to bachelor's degrees. Finally, institutions also offer a limited number of short non-degree awarding postgraduate qualifications.

The information about institutional strategies implemented to sustain international enrolment and to support existing international students during the COVID-19 pandemic was obtained from 19 semi-structured interviews with top institutional leaders (who define institutional strategies) and middle managers (who operationalise and implement strategies). Interviews were conducted in a sample of 13 institutions illustrative of different institutional profiles and selected for having the largest proportions of international students in their student population. The sample included 4 public universities, 4 private universities, 3 public polytechnics, one private polytechnic and one private institution with both university and polytechnic branches^1^. In most cases interviews were conducted with one person only, but some included both top leaders and middle managers, which explains the participation of 24 individuals in 19 interviews (see Table 2). All interviews were performed by the same researcher, which ensured consistency of approach.

**Table 2.** Interviewees according to institutional profile.

Interviewee	Public University	Private University	Public Polytechnic	Private Polytechnic	Total
Top leader	3	4	4	1	N = 12
Middle manager	3	5	3	1	N = 12

Interviews were conducted between November 2020 and April 2021 in the context of a larger project about institutional strategies to promote international enrolments. In this study, however, only responses related to the pandemic were analysed. Interviewees were prompted to speak about the impact of the pandemic on international students at their institution and about the measures that were put in place to respond to the crisis.

The interviews were conducted complying with the research ethics code of the researcher's institution. At the beginning of the interviews, anonymity and data confidentiality were guaranteed by the interviewing researcher and interviewees were informed about the recording of the interviews. Participants also signed an informed consent.

Interviews were fully transcribed and the data was organised and coded by the authors through the software *MaxQDA* according to a grid of categories that emerged during the data analysis. A thematic content analysis technique (Silverman, 2001; Tonkiss, 2006) was employed. This grid contained two main dimensions of analysis – *recruitment strategies* and *mitigation strategies* – each consisting of several categories organised by different themes of analysis.

## Findings

### Impact of COVID-19 on the Number of Enrolments

**Figure 1.**
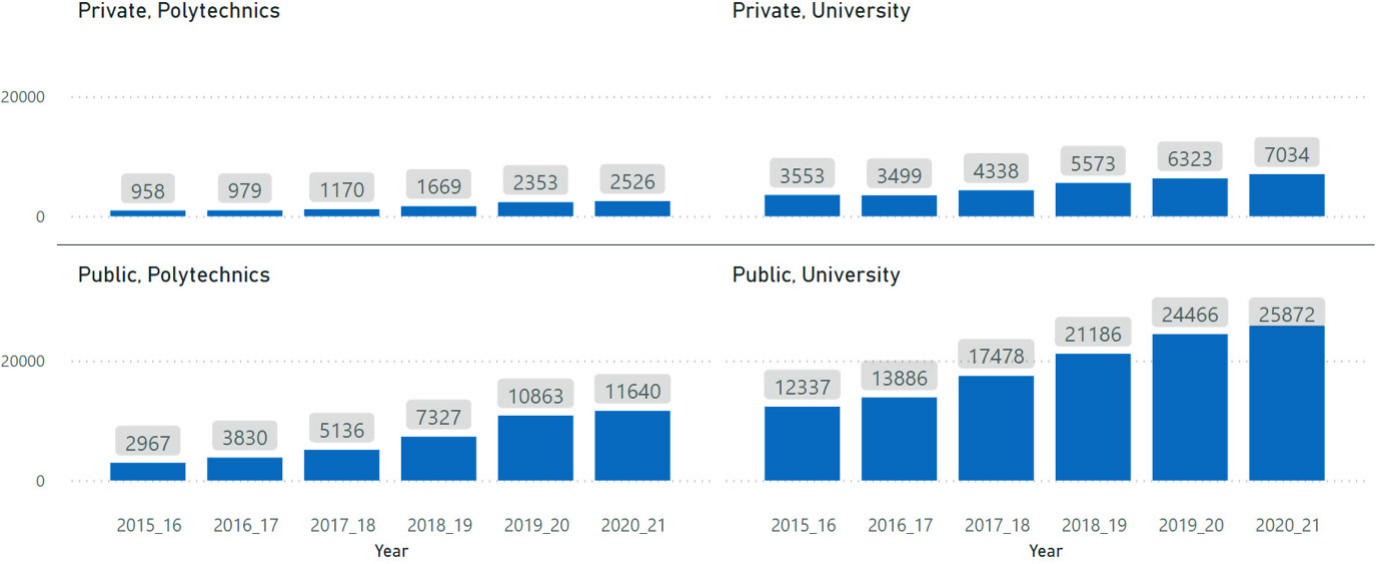
Evolution of international enrolments by institution type.

The absolute numbers for 2020/2021 show an increase in the number of enrolled students in comparison to 2019/2020 (see Figure 1).

**Figure 2.**
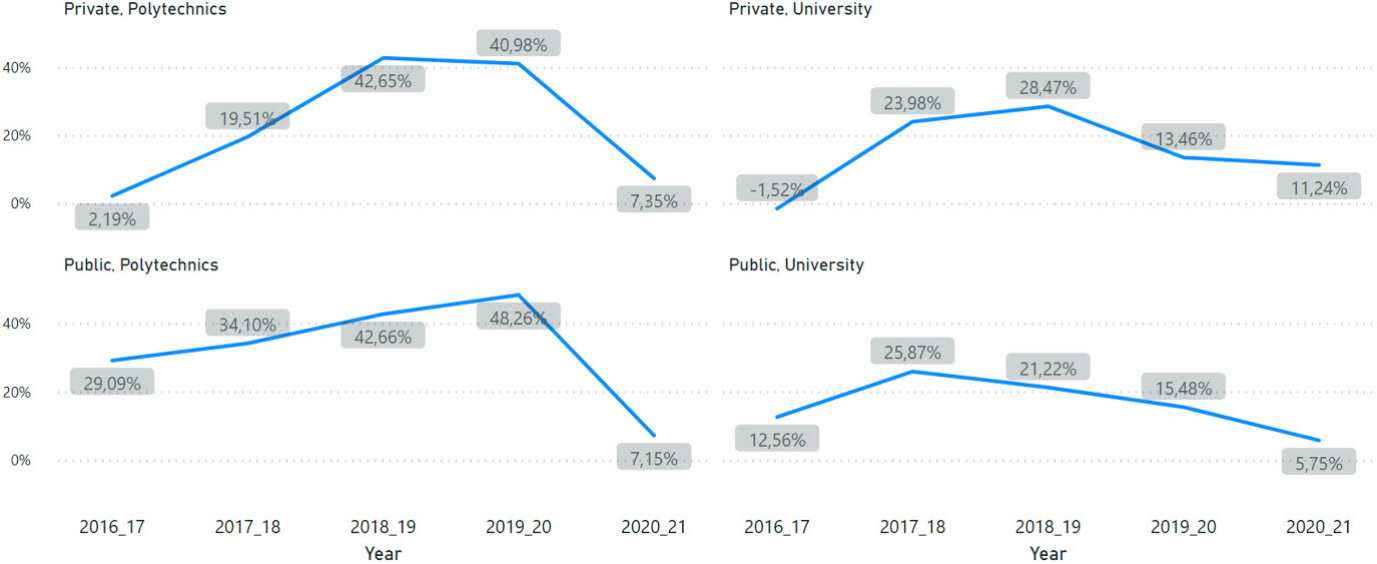
Variation in growth rate (%) of international enrolments.

However, the growth rate of enrolments deaccelerated. Figure 2 shows the percentage of the variation for each HEI subsector calculated through the difference between the number of enrolments registered in a year N and N  +  1. The lower rate of growth is more visible in the case of polytechnic institutions. For example, while international enrolments were growing by around 50% in public polytechnics in the two previous years, the growth rate of the last year was only 7.15%.

Although, on the positive side, Portuguese institutions did not see a reduction in absolute enrolments in relation to the previous years, the steady pace of growth in the years before the pandemic was not sustained, especially in the type of institutions with great growth potential (polytechnic institutions) given their lower attractiveness for national students. The pandemic may be responsible for this deceleration, but other global developments may equally have played a role (e.g. South-South migration).

### Impact of COVID-19 on Institutional Strategies

The interviewees in the selected institutions recognised the impact of COVID-19 on enrolments. While at the stage of applications there was little impact, fewer students afterwards decided to effectively enrol. However, several stated that the decrease in enrolments was less severe than they had expected, which is verified in the national statistics presented above. Interviewees highlighted delays in visa processing times, travel difficulties and border closures as obstacles for students to decide studying in Portugal. To manage the challenges posed by COVID-19, institutions acted at two different levels. First, they revised institutional strategies for international recruitment targeted at potential candidates. Second, they implemented mitigation strategies to reduce the negative impact of the pandemic on current international students.

Regarding recruitment strategies, given the impossibility of meeting potentially interested individuals face-to-face, they invested in virtual tools to advertise the institution and its offer: virtual fairs, virtual visits to the campus, virtual meetings with interested candidates, chatbots, social networks etc. The representative of a public polytechnic illustrated this new approach as follows:

We have been adjusting, our student support services have a chatbot and so we are better connected today to international students than before… There has been an investment in this proximity network, although online and remote, but not only through Facebook and Instagram, so all the social networks have also been a big investment, we have been trying to make up for the lack of face-to-face contact with our presence through digital media.

Additionally, adjustments were made to the application timings to accommodate especially Brazilian students who took the secondary education exams later. Candidates were also offered the possibility of conducting online admission exams.

The attractiveness of Portugal was also associated with the perception of a good management of the pandemic in the country and a less severe situation than elsewhere (for example United States and Brazil). Some interviewees actually noted an increase in enrolments from European and Brazilian students, which may be due to the dramatic situation that their countries experienced regarding the management of the pandemic. At the same time, Portugal was not heavily affected at the beginning of the pandemic, which may have created a sense of safety to these students. A top manager in a private university referred to the higher enrolments of Brazilian students in first degrees in contrast with previous years, when they sought mostly postgraduate degrees:

I think this is related to the particularly difficult situation that Brazil has experienced with the pandemic. And so, who could afford to send their sons and daughters to study abroad did so. It is not usual for us to have so many Brazilians in first cycles, neither students who are so young coming from Brazil. So, I think it is really because of the current situation in Brazil. So, and not wanting this to sound bad, we can say that in general terms the impact of the pandemic has been positive for us.

Concerning the mitigation strategies to reduce the negative impact of the pandemic, these were implemented at three levels: student support, teaching/learning adjustments and safety measures. However, the content analysis performed on the interviews revealed that student support measures were those which received greatest priority among the selected institutions. Support included assistance with logistical matters, peer-support structures, financial support, psychological support and other kinds of generic guidance. Logistical support was by far the most frequently mentioned initiative, as illustrated by the joint interview conducted with representatives of a public polytechnic:

We give them food, we give them medicine, we go with them to medical appointments, we pick them up, we take them back home, we speak with their families, we take care of everything. Of course, this is a different experience, isn’t it? Some would say we are lucky, but luck actually requires a lot of work and my team is completely exhausted because ever since this started, they haven’t had a single day off. There are no Saturdays, no Sundays, because it's the way it is.

To alleviate the isolation created by the lockdowns, the interviewees mentioned peer support initiatives promoted by the institutions in order to provide opportunities of interpersonal contact. At the same time, they also described how students themselves were offering mutual support to each other and took the initiative in this respect further to an institutional environment that created a sense of solidarity. A middle manager in a private university describes one such initiative:

One of the initiatives we took was the “second home”, an already existing association, but which was very active during the pandemic. They organised online game nights and even now, for example, when there was a COVID outbreak among Erasmus students, they have been very useful. And they are peers, they feel at ease to talk to each other about things that they wouldn’t talk to us.

Financial support in the form of scholarships or in-kind support (e.g. food, clothes, IT equipment) represented another form of assistance provided to international students in order to minimise the negative impact of the pandemic and ensure that these could continue their studies. Interestingly, this kind of support has only been mentioned by interviewees from polytechnic institutions. For instance, a private polytechnic representative described a situation in which around half of international students did not have computers to attend the courses online and which the institution addressed by helping students to purchase IT equipment at a reduced price. Another example of in-kind support came from a public polytechnic:

We have noted that the international students are the ones who are asking more for help, for different reasons created by the pandemic. For example, Brazilian students have been affected a lot by the currency conversion rates. They may even have enough money there, but with the conversion, here, the situation gets difficult. Angolan students, for example, are not able to receive the money transfers from their parents… we have some situations like these which make it difficult for students and so we are helping them. We have a food and clothing bank.

Psychological support was mentioned in two cases, both in private institutions, in one case offered directly to students who experienced difficulties, and in the other through webinars conducted by professional psychologists intended to make students aware of the psychological effects of isolation. In the case of one institution specialised in health, a team of nurses was set up to monitor international students in isolation and those who contracted covid-19. This support structure was then extended to all students.

In addition to various kinds of support, institutions also acted to adjust teaching and learning to the circumstances imposed by COVID-19. The main concern was to ensure that international students would not be prejudiced. In this sense, interviewees in various institutions mentioned that international students were allowed to attend classes remotely from their countries. The following excerpt from an interviewee in a public university is illustrative of this concern:

We have been trying to make sure that all the students could attend classes from their countries. Even assessment issues have been treated in the different faculties more carefully and differently from the students who… we know that they cannot come to [city] to do the face-to-face exams for the assessment and so we have been trying to solve this.

However, this solution was not feasible for all international students because of the poor internet connections in their home countries. As the literature review showed, African and South American students were most affected by the transition to online teaching due to a lack of availability of digital equipment (Aristovnik et al., 2020). These are the continents of origin of most international students in Portugal.

Remote learning could not work either for students enrolled in Health programmes because of the clinical and practice-based nature of these courses. Therefore, two private institutions with a significant provision of health programmes tried to find alternatives and adjust the academic year's timetable in order to ensure that students would not miss out on the clinical practice component of the course and would manage to graduate. One interviewee stated:

We immediately said: we cannot pass our students without having taken the practical tests. And we waited until the end, and when the lockdown finished, we were able to immediately set in motion the practical classes, either through simulations, or through the real classes in a real clinical work environment with patients. (…) We extended the academic year until September so that all the students were able to attend the minimum mandatory practical classes, either in a lab environment or in a real work environment. So, we saved the year and I can tell you that students in the 5^th^ year, which is exclusively clinical practice, are finishing their theses now.

An interviewee from a private university referred to the decision of keeping post graduate degrees in a face-to-face mode at the beginning of the 2020/21 academic year, because most international students were enrolled in these qualification levels. This approach represented also a means of alleviating the isolation international students could feel and recognising that teaching also plays a socialising role.

## Discussion and Conclusion

The aim of this paper was to analyse whether Portuguese higher education institutions approached the pandemic as a threat or as an opportunity. Statistical data show that COVID-19 has had a negative impact on degree enrolments of international students, although not as severe as expected. The country's management of the crisis may have prevented a more severe decline in student numbers (Marginson 2020), and may even have contributed to an increase in the case of students from Brazil, a country which suffered dramatically with the government's attitude towards the pandemic. The absolute numbers of enrolments were higher than in the previous year, although the growth occurred at a smaller pace. Following the upward trend since 2015/16, the number of students grew at all levels of education except for postgraduate specialisations. The analysis by type of HEI showed that the slowdown in growth was much more pronounced in polytechnics than in universities, which might indicate that the pandemic has represented more of a threat for the former than for the latter. This may be a consequence of the pattern of growth and the international publics of polytechnic institutions. Compared to universities, international enrolments in polytechnic institutions grew at a much higher pace in previous years, because of their lower attractiveness for national students and the need to fill vacancies with international students. Moreover, a large share of international students, in public polytechnics particularly, come from African Portuguese-speaking countries (Sin et al., 2021), which have been more affected by visa delays and travel restrictions than European countries, for example. African students have also experienced more severe financial hardships during the pandemic (Aristovnik et al., 2020).

However, higher education institutions seem to have been fast in reacting positively to this situation and seem to have faced the challenges brought by the pandemic as an opportunity to rethink their traditional recruitment strategies, for instance, by targeting more European students, who are closer to Portugal and who are unlikely to change travel plans because of visas, travel bans and other bureaucratic problems. This may constitute one of the reasons why a positive result was observed in the absolute number of enrolments, which, as some mentioned, was higher than expected. Additionally, they switched to digital strategies to advertise themselves and their educational offer.

Regarding institutional strategies to mitigate impact on international students already enrolled in Portugal during the pandemic and to support them, a difference was noted by type of institution. While the analysed universities mentioned mainly strategies in terms of teaching adjustments during the transition to distance education, similar to those in other countries (Agasisti & Soncin, 2021; de Boer, 2021; Pham & Ho, 2020), polytechnics referred more to student support, especially at non-academic level, such as logistical and financial assistance, as well as access to essential goods. It should be noted that although most strategies mentioned during the interviews targeted international students, some support measures applied to the whole student body. The differentiation in terms of strategies may reflect the socioeconomic inequalities that exist between students in both types of education, with universities generally being more elitist and polytechnics having a student body more representative of the student population as a whole (Sá et al., 2021). The broad support measures mentioned by the analysed polytechnics may also reflect the stronger dependence on international students to ensure sustainability, given polytechnics’ lower attractiveness for domestic students, especially in locations outside the big metropolitan areas of Lisbon and Porto.

One could therefore argue that the analysed Portuguese higher education institutions have faced COVID-19 as an opportunity to rethink actions and define strategies. However, instead of merely adopting a “crisis management” approach, HEIs would benefit from medium-long-term planning and sustainable transformation and, based on lessons learned, embed some of the practices adopted during the COVID-19 period in their day-to-day activities in the future. This study has been exploratory, and its findings need further corroboration with more institutions, especially polytechnic ones, since these latter have been more difficult to recruit as participants in the study. Another limitation is the missing perspective of international students, so it is also important in future research to get insight into their point of view to understand whether the actions implemented by HEIs during the pandemic were satisfactory and effectively met their needs.

## Notes

1. Following the institutional accreditation in 2017, the Portuguese HE system now comprises 102 higher education institutions: 16 public universities (including 2 military and police institutions and the Open University), 22 private universities, 20 public polytechnics and 44 private polytechnics. Most private polytechnics are small, specialised institutions.
